# Tracts on Generation

**Published:** 1848-04

**Authors:** 


### EDITORIAL DEPARTMENT.

---

*Tracts on Generation*; Translated from the German, by C. R. Gilman, M. D., Prof. of Obstetrics, &c., College of Physicians and Surgeons, New-York; and Theodore Tellkampf, M. D., Gefhard Professor, Columbia College. New-York, Samuel S. and Wm. Wood, 271 Pearl St. 1847, 8vo. p.p. 65.

This is the first of a series of publications on the subject of generation proposed to be issued, if the publishers meet with sufficient encouragement. It is a translation of a work thus entitled:—"Proofs that the periodic maturation and discharge of ova are, in the mammalia, and the human female, independent of coition, as a first condition of their propagation. By T. L. G. Bischoff, M. D., Prof. of Physiology, &c., Giessen."

Readers of medical periodicals are already acquainted with the new views of ovulation and fecundation which have been advanced by Bischoff, and other German physiologists. These views are compendiously expressed in the title page just quoted. They are not only highly interesting in a scientific point of view, but, if established, will be found to lead to various important practical deductions. One highly important relation, which may be mentioned, concerns medico-legal questions turning on the precise period of conception. Other bearings will at once suggest themselves to the mind of the reader. A detailed account of the proofs upon which these new views are based, must, therefore, prove highly acceptable to those members of the profession who are alive to progressive developments in the several departments of our science.

Without professing to have given to the evidence adduced that careful, deliberate attention which the subject merits, we are free to say that the proofs of the periodic maturation and discharge of ova of the mammalia, exclusive of the human female, seems to us pretty conclusively established. The argument of analogy, as respects the human female, it must be confessed, is strong; still, the position that the menstrual period is coincident with the maturation and discharge of ova, and analogous to the period of heat in inferior animals, seem to lack positive proof. With reference to this point, it is desirable that facts should be gathered bearing on the question whether fecundation be not known to occur at times sufficiently remote from the menstrual period to disprove the hypothesis that ova are produced exclusively at that period.

The present publication is on good paper, with large clear type, and is creditable alike to Translators and publishers. We trust the profession will sufficiently appreciate the value of a series of publications of which this is the commencement, to afford the encouragement requisite to insure its continuation.

